# Emergence of vancomycin-resistant *Enterococcus faecium vanA* ST612 with reduced daptomycin susceptibility, Switzerland, 2018 to 2024

**DOI:** 10.2807/1560-7917.ES.2025.30.45.2500227

**Published:** 2025-11-13

**Authors:** Danielle Vuichard-Gysin, Andrea C Büchler, Dominique S Blanc, Peter M Keller, Pascal Schläpfer, Andreas Kronenberg, Vanja Piezzi, Patrice Nordmann, Laurence Senn, Stephan Harbarth, Sarah Tschudin-Sutter

**Affiliations:** 1 Swiss National Centre for Infection Prevention, Swissnoso, Bern, Switzerland; 2 Department of Infectious Diseases and Infection Prevention, Thurgau Hospital Group, Frauenfeld and Münsterlingen, Switzerland; 3 Infection Prevention and Control Unit, Infectious Diseases Service, Lausanne University Hospital and University of Lausanne, Lausanne, Switzerland; 4 National Reference Centre for Emerging Antibiotic Resistance (NARA), Fribourg, Switzerland; 5 Clinical Bacteriology and Mycology, University Hospital Basel, Basel, Switzerland; 6 Laboratory Medicine, University Hospital Basel, Basel, Switzerland; 7 Institute for Infectious Diseases, University of Bern, Bern, Switzerland; 8 Federal Office of Public Health, Bern, Switzerland; 9 Medical and Molecular Microbiology, University of Fribourg, Fribourg, Switzerland; 10 Infection Control Programme, Geneva University Hospitals and Faculty of Medicine, WHO Collaborating Center, Geneva, Switzerland; 11 Division of Infectious Diseases, University Hospital Basel, Basel, Switzerland

**Keywords:** VRE, daptomycin non susceptibility, ST612, emergence, outbreak, risk factor

## Abstract

We describe the emergence of vancomycin-resistant *Enterococcus faecium* (VREfm) *vanA* ST612 in Switzerland from 2018 to 2024 that resulted in a national outbreak investigation. This clone has predisposing genetic alterations associated with reduced daptomycin susceptibility. The National Nosocomial Outbreak Investigation Center was commissioned to assess the temporospatial distribution of this clone in Switzerland and evaluate its clinical impact. Core genome multi-locus sequence typing (cgMLST) revealed five separate VREfm *van*A ST612 clusters of different sizes across different healthcare regions, but predominantly in the German-speaking part. The broad geographic dissemination and temporal variation in detection suggests multiple introductions to the healthcare system. One of these cgMLST clusters (n = 79 cases) with an infection rate of 12.8% was ongoing, mainly affecting patients with extensive contact to the Swiss healthcare system or prior antibiotic exposure. The detection of daptomycin non-susceptibility in patients without prior daptomycin exposure suggests ongoing *E. faecium* adaptation due to external pressures. Future prevention efforts should emphasise assessing barriers for active surveillance cultures, developing a national standard for cost-effective sequencing methods and promoting the sharing of sequencing results together with epidemiological metadata. Our report intends to raise awareness as this sequence type might already be spreading undetected in European countries.

**Key public health message**

**What did you want to address in this study and why?**

We looked at an antibiotic-resistant and hard to treat bacterium vancomycin-resistant *Enterococcus faecium* (VRE) which spreads in hospitals and can cause severe infections in critically ill patients and those with a weakend immune system. In early 2024, a new and even more antibiotic resistant type of VRE (VREfm *vanA* ST612) was discovered in Switzerland. It quickly spread between hospitals and regions leading to a national investigation.

**What have we learnt from this study?**

We found that patients who had close (and repeated) contact with the Swiss healthcare system or had taken antibiotics recently were more likely to acquire these resistant bacteria. Many cases likely went unnoticed because hospitals were not actively checking for VRE in patients. This may have allowed it to spread silently.

**What are the implications of your findings for public health?**

Clinicians and microbiologists should be aware of this new VRE type, which could be harder to treat, and might already be spreading in other countries without being detected. Public health efforts should focus on understanding what prevents hospitals from fully applying infection control measures, facilitating cost-effective testing methods and encouraging the sharing of results to help stopping the spread.

## Background

Vancomycin-resistant Enterococcus (VRE) is listed as a high priority pathogen for research and development and infection control measures by the World Health Organization (WHO) [1]. Over the past decade, VRE have emerged in hospitals, causing outbreaks and severe infections in immunocompromised patients [2,3]. In Europe, prevalence of vancomycin-resistant *Enterococcus faecium* (VREfm) infections in healthcare settings is on the rise. In the latest (2022–2023) European Centre for Disease Prevention and Control (ECDC) point prevalence survey on healthcare-associated infections and antimicrobial use in acute care hospitals, vancomycin resistance was reported in 15.6% of isolated enterococci as compared to 10.8% in the previous survey (2016–2017) [4,5]. In contrast, the prevalence of VREfm still remains low in Switzerland (2.2% in 2023) [6]. Distinct genotypes of vancomycin resistance exist (*vanA*, *vanB*, *vanC*, *vanD*, and *vanE*) with *vanA* and *vanB* being the most prevalent and clinically significant types for humans [7]. While VREfm *vanA* isolates of sequence type (ST) 612 are the third most common ST in France, it seems uncommon in other European countries beyond local outbreaks [8-12]. Vancomycin resistance in *E. faecium* limits treatment options in case of infection and is associated with higher mortality and healthcare costs compared with infections caused by vancomycin-susceptible enterococci [13]. Daptomycin is commonly used for treatment of invasive VREfm infections. However, treatment success with this compound depends on the dosage and minimum inhibitory concentration (MIC) [14]. Prevalence of daptomycin resistance is currently not reported in the European Antimicrobial Resistance Surveillance Network (EARS-Net) database due to a missing European Committee on Antimicrobial Susceptibility Testing (EUCAST) breakpoint [15].

### Outbreak detection

In early 2024, the molecular diagnostics laboratory of a university hospital in north-western Switzerland (NWS laboratory) noted an increase in closely-related VREfm *vanA* ST612 isolates originating from various healthcare institutions across five of the 26 cantons in Switzerland. Identification of two mutations known to be associated with decreased daptomycin susceptibility caused additional concern and prompted a nationwide investigation on behalf of the Swiss Federal Office of Public Health (FOPH). A first situation analysis took place on 29 January 2024 between the FOPH and the National Nosocomial Outbreak Investigation Center (NOIC), which is operated by Swissnoso, the Swiss National Center for Infection Prevention. A task force consisting of representatives from Swissnoso, the National Reference Laboratory for Emerging Antimicrobial Resistance (NARA), and the FOPH was subsequently established and a second meeting was held on 7 February 2024. In this meeting the experts evaluated the need for an in-depth epidemiological analysis and discussed the most pressing steps, such as immediate release of recommendations for preventive measures and a communication strategy. Based on the available epidemiological, microbiological and molecular data, the objectives of further investigations were as follows: (i) estimate the prevalence of VREfm *van*A ST612 in 2024, (ii) assess the temporospatial distribution of this clone in Switzerland and identify the chains of transmission in order to implement effective control measures, and (iii) describe clinical characteristics, morbidity and mortality.

We describe the emergence of a clone of VREfm *van*A ST612, which has rarely been identified in Europe outside France and exhibits reduced daptomycin susceptibility, affecting several healthcare networks in various regions of Switzerland between November 2018, when VREfm *vanA* ST612 was first detected, and April 2024. We intend to raise awareness regarding this clone and its clinical implications as treatment success of invasive infections may be compromised.

## Methods

### Study design

This is a retrospective outbreak report triggered by the results of an ad hoc planned outbreak investigation using clinical and molecular-based epidemiological data. We included all patients in whom a clinical specimen or screening sample was positive for VREfm *vanA* ST612 from when it was first identified in Switzerland in November 2018 until April 2024.

### Data sources and case definitions

**Figure 1:**
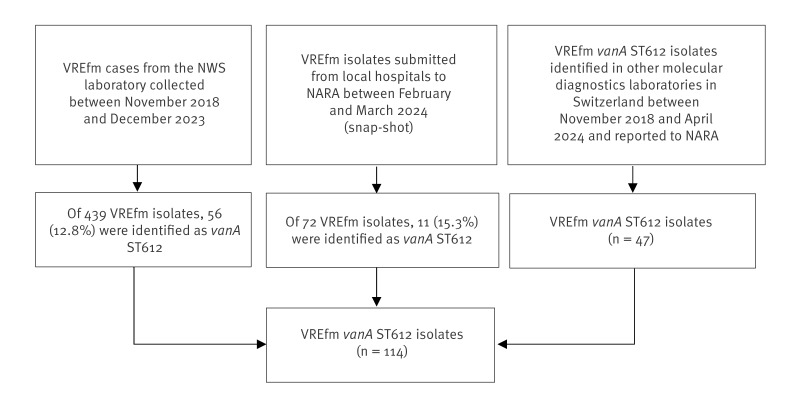
Composition of the total number of vancomycin-resistant *Enterococcus faecium* *van*A ST612 isolates available for epidemiological investigation, Switzerland, 2018–2024

NARA: the National Reference Laboratory for Emerging Antimicrobial Resistance; NWS: the molecular diagnostics laboratory of a university hospital in north-western Switzerland; ST: sequence type; VREfm: vancomycin-resistant *Enterococcus faecium.*

Cases of VREfm *van*A ST612 were prospectively and retrospectively identified based on the collections of isolates from the NWS laboratory and the NARA. All other laboratories in Switzerland performing whole genome sequencing (WGS) were encouraged to send sequencing data of previously identified VREfm *van*A ST612 to the NARA to enable centralised comparisons by core genome multi-locus sequence typing (cgMLST). From February to March 2024, all hospitals were requested to send newly identified VRE isolates to NARA for further analysis (Figure 1). Genomic data were shared between sequencing laboratories and the NARA along with the initials and date of birth of the respective patients, which ensured minimal risk of duplicate isolates. One isolate per case was included.

The NOIC contacted all hospitals with known cases of VRE *vanA* ST612 and asked them to enter anonymised epidemiological and health-related data of their sporadic cases, index and secondary cases of local clusters as well as information on contact investigations into an electronic data registry. The definitions of cases and clusters are listed in Table 1.

**Table 1:** Definitions of cases and clusters of vancomycin-resistant *Enterococcus faecium* *van*A ST612 applied during our outbreak investigation, Switzerland, 2018–2024

Case	Patient with carriage^a^ of VREfm *vanA* ST612 between 2018 and 2024
Sporadic case	Patient with carriage of VREfm *vanA* ST612 without any spatiotemporal^b^ link to another carrier of VREfm *vanA* ST612 within a healthcare institution
Local cluster	A cluster of VREfm *vanA* ST612-cases was defined as two or more patients with carriage of VREfm *vanA* ST612 with a spatiotemporal^b^ link within a healthcare institution
Index case	Patient with carriage of VREfm *vanA* ST612 considered the first patient within a local cluster
Secondary case	Patient with carriage of VREfm *vanA* ST612 detected within a contact investigation after an index case was detected
cgMLST cluster	Clustering isolates with < 4 loci differences between them in the cgMLST analysis

cgMLST: core genome multi-locus sequence typing; ST: sequence type; VREfm: vancomycin-resistant *Enterococcus faecium.*

^a^ Carriage refers to colonisation or infection.

^b^ The definitions varied depending on the individual hospitals.

In addition, the database of the Swiss National Antibiotic Resistance Centre (ANRESIS), covering 90% of all Swiss acute care hospitals, was searched for all VRE isolates from clinical cultures and screening samples submitted between November 2018 and April 2024. Sequence typing was not available for this dataset.

### Epidemiological investigation

The NOIC contacted the infection prevention and control (IPC) teams of the respective hospitals where isolates of VREfm *vanA* ST612 were identified. The IPC teams were asked to fill in an electronic case report form using REDCap electronic data capture tools [16,17].

We collected the following data: the hospital in which the patients were staying when VREfm *van*A ST612 was first detected in a specimen; patient information (age, sex, date of admission, ward, main underlying disease, treating specialty and interventions within 3 months before first detection, directly transferring institution, type of institutions the patient had stayed in or regularly visited within 3 months before VRE detection, antibiotic use within 3 months prior to VRE detection, type of infection with VRE, antibiotic treatment for, and outcome of, VRE infection); microbiological information (date of first detection, type and body site of first positive isolate, MIC and method for susceptibility testing for daptomycin and linezolid, analysis by WGS, additional positive body sites, date of last detection, carriage of another multidrug-resistant organism (MDRO)); suspected or confirmed source of the VREfm *van*A ST612 isolate; definition used for contact patients; number of identified and screened contact patients; number of secondary cases; performance of ward-wide screenings including frequency and duration.

If a cluster or an outbreak occurred in a hospital, we asked for the following additional information: start date of the cluster/outbreak; when the outbreak was contained; end date of the outbreak; how the end of the outbreak was determined; date of last case detected within the cluster/outbreak; number and description of affected wards; probable source of cluster/outbreak; most likely path of transmission to secondary cases; date of first reporting to cantonal physician; identification of patient as index or secondary case.

### Microbiological investigation

Identification of VRE was performed by the individual microbiology laboratories of the affected hospitals following their individual standard operation procedures including standard antibiotic susceptibility testing.

For all VRE isolates received by the NARA between February and March 2024, antimicrobial susceptibility testing for vancomycin, teicoplanin, quinupristin/dalfopristin, tetracycline, daptomycin, ciprofloxacin, erythromycin, tigecycline, linezolid, gentamicin, ampicillin and chloramphenicol was performed in broth microdilution (EUVENC Sensititre, ThermoFisher Diagnostics, Dardilly, France). The EUCAST breakpoint table version 14.0 [15] was used to interpret antimicrobial susceptibility testing results. For those VREfm isolates obtained between February and March 2024, sequencing of the *gyd* gene (one of the seven MLST genes) was performed, and WGS was subsequently conducted on all isolates with *gyd* allele #5 using an Illumina platform (Illumina, San Diego, United States (US)).

Sequences of previously identified VREfm *van*A ST612 from hospitals that perform sequencing as part of regular VRE surveillance were directly included in the WGS analysis. Genomic comparisons were performed by cgMLST using SeqSphere version 10.0, (Ridom, Muenster, Germany). The cgMLST schema consisted of 1,340 loci of the core genome. Based on the spatiotemporal distribution of Swiss ST612 isolates, a cut-off of < 4 loci difference was considered adequate for defining cgMLST clusters [18].

Analysis of the resistome (ResFinder [19]) was performed on all sequenced isolates.

### Statistical methods

We applied descriptive statistics using IBM SPSS Statistics version 28.0.1.0 (IBM Corp., Armonk, US). Continuous variables were expressed as medians and interquartile ranges and categorical variables as frequencies and percentages.

## Results

### Epidemiological and microbiological investigation

Overall, 114 cases of VRE *van*A ST612 were identified between November 2018 and April 2024. These cases were obtained from the collection of samples by the NWS laboratory initially triggering the outbreak investigation, the retrospectively reported cases by hospitals and laboratories performing sequencing and the isolates identified by the NARA during the intensified screening period from February to March 2024 (Figure 1).

**Figure 2:**
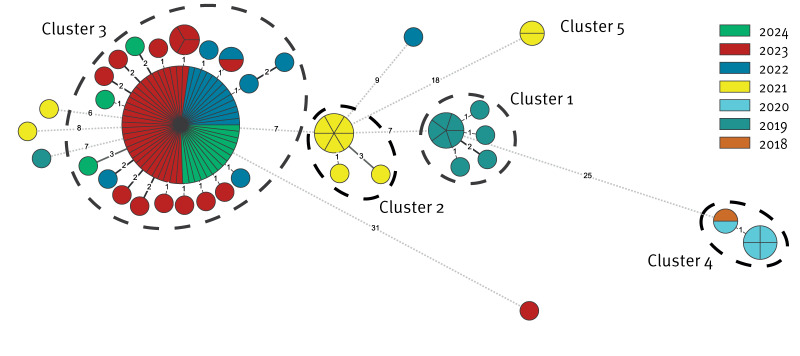
Core genome multi-locus sequence typing minimum spanning tree of vancomycin-resistant *Enterococcus faecium* *vanA* ST612 isolates, Switzerland, 2018–2024 (n = 110^a^ 96.5%)

^a^ The total number of isolates was 114, however, four isolates were not available for analysis.

From 2018 to 2024, cgMLST revealed five separate clusters, each including 2–79 cases with fewer than four loci differences differences and six non-related cases (Figure 2).

The colours of the isolates represent the year of isolation. Each coloured circle represents one or more isolates. The numbers between circles indicate the loci distance between isolates. The five clusters of genetically closely related isolates are indicated by dashed circles.

**Figure 3:**
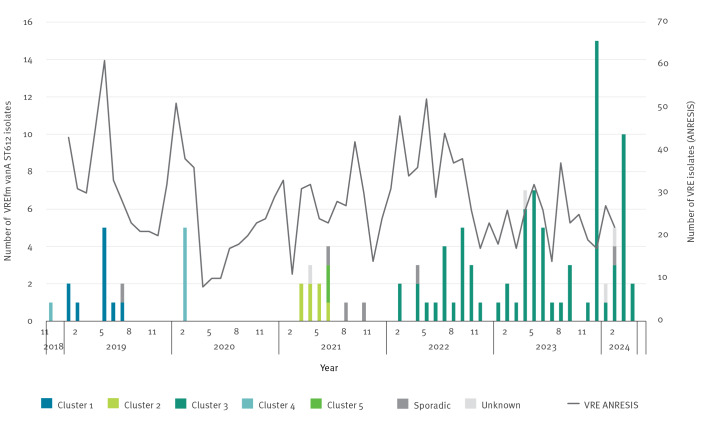
Epidemiological timeline of vancomycin-resistant *Enterococcus faecium* *vanA* ST612 cases and clustering according to core genome multi-locus sequence typing compared with vancomycin-resistant *Enterococcus* species cases submitted to the database of the Swiss National Antibiotic Resistance Centre, Switzerland, 2018–2024

ANRESIS: the database of the Swiss National Antibiotic Resistance Centre; ST: sequence type; VRE: vancomycin-resistant *Enterococcus* species; VREfm: vancomycin-resistant *Enterococcu faecium.*

**Figure 4:**
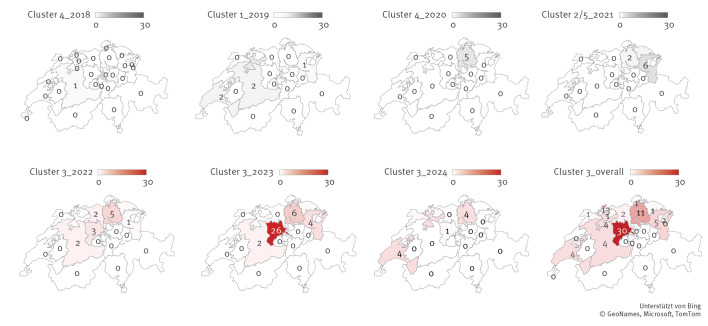
Spatiotemporal distribution of the five core genome multi-locus sequence typing clusters of vancomycin-resistant *Enterococcus faecium* *vanA* ST612 cases detected across the cantons of Switzerland, 2018–2024

Cluster 1 appeared in 2019 and includes 10 isolates from six hospitals in six different cantons from south-western to the north-western and the north-eastern part of Switzerland. Cluster 2 and Cluster 5 both appeared independently from each other in 2021, affecting one hospital in the French-speaking part and five hospitals in the German-speaking part of Switzerland. The earliest case of Cluster 4 was seen in 2018 and the cluster includes six isolates detected in two hospitals in two different cantons. The latest cases in this cluster were detected in February 2020. Cluster 3, the largest of the five clusters with a predominant accumulation in central Switzerland, was first detected in 2022. Until April 2024, it included 79 closely related isolates originating from 13 of the 26 Swiss cantons, mainly in the northern Geman-speaking region. One canton detected 30 (38%) VREfm *vanA* ST612 cases during this time period (2022–2024). No isolates originating from the Italian-speaking part of Switzerland were identified during our study period. The epidemiological timeline with colour-coding for the respective cgMLST clusters is shown in Figure 3 and the geographical distribution over time in Figure 4. There was no increase seen in VRE isolates registered by ANRESIS between 2018 and 2024, suggesting that STs other than ST612 dominated in these years (Figure 3).

In grey from left to right, spatiotemporal distributions of cgMLST Clusters 1, 2, 4, and 5 per year (2018–2021) are shown. In red, spatiotemporal distribution of the cgMLST Cluster 3 per year (until April 2024) and overall distribution is shown.

Analysis of the resistome using ResFinder [19] across 110 sequences revealed that all initially detected VREfm *van*A ST612 isolates harboured the mutations W73C in *liaR* and T120A in *liaS*, both of which are associated with reduced susceptibility to daptomycin [20,21]. Additionally, all sequences carried resistance-associated mutations including *aac(6')-I* (amikacin, kanamycin), *msr(C)* (aminoglycosides) and *ant(6)-Ia* (quinolone), as well as the *vanA* gene. Reduced daptomycin susceptibility was further supported by the MIC testing results: all VREfm *vanA* ST612 isolates (n = 11) submitted to the NARA between February and March 2024 exhibited a consistent MIC of 4 mg/L, whereas MIC values varied among other sequence types (Supplementary Figure S1 shows the distribution of daptomycin MICs for the VREfm *vanA* ST612 isolates compared to the other VRE isolates submitted during the intensified screening period from February to March 2024). Minimum inhibitory concentration values in microdilution also revealed that all VREfm *van*A ST612 isolates were resistant to vancomycin (> 128 mg/L), teicoplanin (16–32 mg/L), ciprofloxacin (> 16 mg/L), erythromycin (> 128 mg/L) and ampicillin (> 64 mg/L) and had high-level resistance to gentamicin (> 1,024 mg/L). All isolates were susceptible to quinupristin/dalfopristin (1 mg/L), tigecycline (0.06 to 0.25 mg/L) and linezolid (2 mg/L).

### Investigation of ongoing (at the time of the study) outbreak of core genome multi-locus sequence typing Cluster 3 cases

Since ongoing transmission was observed in cases belonging to cgMLST Cluster 3 (n = 79), an in-depth outbreak investigation was commissioned by the FOPH for this specific cluster. For 78 of the 79 cases in cgMLST Cluster 3, individual patient data were available (Table 2). Direct transfer from a Swiss healthcare institution was reported for 15 (45.5%) sporadic cases and three (37.5%) index cases of local clusters. Contact with the Swiss healthcare system within 3 months prior to first detection was reported for 28 (84.8%) sporadic cases and six (75%) index cases of local clusters. Antibiotic exposure within 3 months was frequently reported for both sporadic (90.2%) and index (87.5%) cases. Prior daptomycin treatment, however, was rare and only reported in secondary cases (n = 2, 2.6%).

**Table 2:** Epidemiological information and characteristics of vancomycin-resistant *Enterococcus faecium* Cluster 3 patients according to core genome multi-locus sequence typing, Switzerland, 2022–2024

Epidemiological information and patient characteristics	All cases (n = 78)^a^		Sporadic cases and index cases of local clusters (n = 41)		Sporadic cases (n = 33)		Index cases of local clusters (n = 8)		Secondary cases of local clusters (n = 37)	
Source information and healthcare exposure	n	%	n	%	n	%	n	%	n	%
Geographical distribution^b^										
German-speaking part	73	93.6	36	87.8	27	81.8	8	100	37	100
French-speaking part	5	6.4	5	12.2	5	15.2	0	0	0	0
Probable source										
Other healthcare institution	10	12.8	10	24.4	10	30.3	0	0	NA	NA
Repatriation from abroad	1	1.3	1	2.4	1	3	0	0	NA	NA
In-hospital transmission overall	37	47.4	NA	NA	NA	NA	NA	NA	37	100
In-hospital transmission: roommate	8	10.3	NA	NA	NA	NA	NA	NA	NA	NA
In-hospital transmission: ward-wide SCR	20	25.6	NA	NA	NA	NA	NA	NA	20	54.1
In-hospital transmission: other link	3	3.8	NA	NA	NA	NA	NA	NA	3	8.1
In-hospital transmission: unknown link	7	7.7	NA	NA	NA	NA	NA	NA	6	16.2
Unknown	29	38.5	29	73.2	22	66.7	8	100	8	21.6
Direct transfer from another healthcare institution										
Other hospital in Switzerland	24	30.8	18	43.9	15	45.5	3	37.5	6	16.2
Long-term care facility in Switzerland	3	3.8	2	4.9	2	6	0	0	1	2.7
Other hospital abroad	1	1.3	1	2.4	1	3	0	0	0	0
No direct transfer	48	61.5	20	48.8	15	45.5	4	50.0	29	78.4
Unknown	2	2.6	0	0	0	0	1	12.5	1	2.7
Previous treatment in another healthcare institution in Switzerland^c^										
Any Swiss healthcare institution	57	73.1	33	82.5	28	84.8	6	75	23	62.2
Same Swiss healthcare institution as first detection	38	48.7	18	43.9	15	45.5	3	37.5	20	54.1
Other Swiss healthcare institution	25	32.1	21	51.2	18	54.5	3	37.5	4	10.8
Swiss long-term care facility	4	5.1	3	7.3	3	9.1	0	0	1	2.7
None	21	26.9	7	17.1	5	15.2	2	25.0	14	37.8
Abroad	1	1.3	1	2.4	1	3	0	0	0	0
Contact investigations	n	%	n	%	n	%	n	%	n	%
Definition of contact patients										
Roommates	NA	NA	17	41.5	15	45.5	2	25.0	NA	NA
Roommates/same bathroom	NA	NA	5	12.2	3	9.1	2	25.0	NA	NA
Roommates/ward mates	NA	NA	5	12.2	4	12.1	1	12.5	NA	NA
Roommates/same bathroom/ward mates	NA	NA	2	4.9	1	3	1	12.5	NA	NA
Roommates/ward mates/after index	NA	NA	1	2.4	0	0	1	12.5	NA	NA
No contact tracing	NA	NA	10	23.8	9	27.3	1	12.5	NA	NA
Unknown	NA	NA	1	2.3	1	3.0	0	0	NA	NA
Additional ward-wide screenings	NA	NA	12	29.3	9	27.3	3	37.5	NA	NA
Patient characteristics	n	%	n	%	n	%	n	%	n	%
Age at first detection (years), median (range)	72.5	20–97	74	33–97	74	33–97	83	44–85	65	20–88
Sex										
Female	34	43.6	23	56.1	20	60.6	3	37.5	11	29.7
Male	44	56.4	18	43.9	13	39.4	5	62.5	26	70.3
Hospitalisation										
Duration of hospitalisation until first detection in days, median (range)	9.5	0–83	7	0–66	4	0–66	16.5	0–37	12	0–83
Not hospitalised at first detection	2	2.6	0	0	0	0	0	0	2	5.3
ICU stay^b^	24	30.8	13	31.7	9	27.3	4	50	11	29.7
Treating specialty										
Medical	53	67.9	29	70.7	24	72.7	5	32.5	24	64.9
Surgical	25	32.1	12	29.3	9	27.3	3	37.5	13	35.1
Underlying diseases										
Haematological	8	11.5	5	12.2	5	15.2	0	0	4	10.8
Gastrointestinal	28	35.9	17	41.5	13	39.4	4	50.0	11	29.7
Nephrological	28	35.9	18	43.9	15	45.5	3	37.5	10	27.0
Interventions and invasive devices										
Haemodialysis	6	7.7	4	9.8	3	9.1	1	12.5	2	5.4
Long-term urinary catheter	8	10.3	7	17.1	6	18.2	1	12.5	1	2.7
Visceral or urological surgery^b^	12	15.4	5	12.2	3	9.1	2	25	7	18.9
Previous antibiotic therapy^b^	62	79.5	37	90.2	30	90.2	7	87.5	25	67.6
Previous therapy with daptomycin	2	2.6	0	0	0	0	0	0	2	5.4
Other MDRO carriage										
MRSA	3	3.8	1	2.4	1	3	0	0	2	5.4
ESBL non-*Escherichia coli*	4	5.1	3	7.3	3	9.1	0	0	1	2.7
Other MDR Gram-negatives	4	5.1	3	7.3	3	9.1	0	0	1	2.7
None	68	87.2	35	85.4	27	81.8	8	100	33	89.2
Microbiological information	n	%	n	%	n	%	n	%	n	%
Type of isolate of first detection										
Clinical	26	33.3	18	43.9	11	33.3	7	87.5	8	21.6
Screening	52	66.7	23	56.1	22	66.7	1	12.5	29	78.4

ESBL: extended spectrum beta-lactamase; ICU: intensive care unit; MDR: multidrug-resistant; MDRO: multidrug-resistant organism, MRSA: methicillin-resistant *Staphylococcus aureus*; NA: not applicable; SCR: screening.

^a^ There were 79 cases in total. Information was missing for one case.

^b^ In 2022 and 2023, isolates originated only from the German-speaking part.

^c^ Three months before first detection.

Definitions: sporadic case is a case without spatiotemporal link to another patient identified by the healthcare institution; index case is the first case detected within a local cluster; secondary case is a subsequent positive (contact) patient with a spatiotemporal link within a local cluster; local cluster is two or more cases with a spatiotemporal link identified within the same healthcare institution.

While 26 patients (33.3%) had a first detection of VRE in a clinical culture, the treating physicians diagnosed only 10 patients (12.8%) with one or more symptomatic infections upon initial detection or thereafter. These included one bloodstream infection, three surgical site infections, three urinary tract infections and four other types of infections. Four patients were treated with linezolid, two with a combination of daptomycin and linezolid and one with tigecycline. Three patients did not receive any VRE-active treatment. Two patients died from the VRE infection, five died due to other causes and three survived. In the remaining 16 patients, detection of VRE in a clinical culture was interpreted as colonisation and no specific antibiotic treatment was initiated.

The investigation also revealed varying definitions of contact patients among institutions. For sporadic cases, considering only roommates as contact patients was most frequently reported, while broader definitions were applied for index cases within local clusters. Most of these definitions did not align with the Swiss national recommendations and the number of identified and/or screened contact patients was either low or unknown (data not shown) [22].

## Outbreak control measures

In mid-February 2024, the NOIC in collaboration with the FOPH released a first alert to all Swiss healthcare facilities regarding the emergence and spread of VREfm *vanA* ST612 [23]. Based on the sparse epidemiological information available at that time, the alert emphasised strict adherence to the existing national recommendations on VRE screening and issued a temporary recommendation for intensified screening measures for patients being directly transfered from other Swiss acute hospitals, particularly patients from high-risk wards [22,23]. It also reminded hospitals to adhere to the national recommendations for management of outbreaks with multidrug-resistant microorganisms if a local cluster was detected [24,25]. The NARA released a separate but coordinated alert directed at microbiological laboratories focused on the detection of VRE, recommending the susceptibility testing method for daptomycin and the collection of sequencing results of previously identified VREfm *vanA* ST612. Both alerts recommended that an intensified screening strategy should be implemented during the months of February and March 2024, with all newly detected VRE isolates sent to the NARA for further analysis.

By the end of May 2024, updated recommendations for continuing on-admission screening of patients after transfer from, or after repetitive contact with, Swiss healthcare institutions were released [26]. Final recommendations were made in January 2025 with focus on containing the spread of VRE within a healthcare institution [27]; reinforcing compliance with national guidelines on VRE prevention, in particular conducting screening on admission based on local epidemiology; standardising contact investigations following the detection of new VRE cases; ensuring transparent communication within healthcare networks; and complying with mandatory reporting of VRE outbreaks (≥ 3 linked cases) to the cantonal health authorities.

## Discussion

In this outbreak investigation, cgMLST detected a wide distribution of five individual clusters of VREfm *van*A ST612 across different healthcare regions in Switzerland. Ongoing transmission was seen in one of the five cgMLST clusters (Cluster 3), mostly affecting patients with repeated or prolonged contact to the Swiss healthcare system and those with prior antibiotic exposure, which is in line with previous reports [28]. The high proportion of sporadic cases as well as the high rate of detection in clinical samples (44%) in sporadic and index cases of local clusters points to an already wide-spread dissemination with a hidden reservoir of VRE carriers, mostly affecting the German-speaking part of Switzerland. This assumption is further supported by the high infection rate of 12.8% among the cgMLST Cluster 3 cases, which is higher than reported in the literature. A recent meta-analysis found that 8% of VRE-carriers develop infection after a median of 30 days, suggesting that a relevant proportion of patients colonised was not detected [29]. The wide distribution and large temporal differences in detection could be an indicator of multiple introductions into the healthcare system. Suboptimal adherence to prevention and control measures including delayed introduction of active surveillance and inconsistent tracing of contact patients may have facilitated further spread [30].

The MIC for daptomycin of 4 mg/L, found in all tested isolates, has been associated with an increased risk of microbiological failure of daptomycin treatment [31,32]. The increased daptomycin MIC found in our study is probably due to the presence of the two mutations in *liaR* and *liaS*. These mutations are constitutive to all Swiss isolates of ST612. However, genomic determination of daptomycin resistance in *E. faecium* has not been fully elucidated [33]. Development of resistance in relation to daptomycin treatment has previously been reported [34,35]. However, the fact that daptomycin non-susceptibility in patients in our study has been discovered without prior daptomycin exposure may indicate an ongoing evolution of *E. faecium* due to external challenges [36-38] and transmission of resistant isolates between patients [39], which could pose an even greater clinical and public health challenge.

Awareness is therefore needed as the clone might already be circulating undetected within other European countries. Associated reduced daptomycin susceptibility may be missed depending on the method used for susceptibility testing. This may challenge treatment success in infected patients, potentially leading to higher mortality [31,40].

Vancomycin-resistant *E. faecium* *vanA* ST612 has previously been described in Europe with the highest prevalence in France, a country with low VRE endemicity [12]. While vancomycin resistance in *E. faecium* remains low in Switzerland (2.2% in 2023), there has been a notable increase in VREfm colonisations and infections over the past decade, primarily due to nosocomial spread [6,28,41,42]. In the present study, the combination of individual clinical and genomic metadata from several healthcare institutions has shown that a VREfm *van*A ST612 clone has disseminated across several healthcare facilities and regions in Switzerland. Application of cgMLST has been shown to outcompete MLST when investigating healthcare associated VRE outbreaks [43]. Furthermore, it facilitates interlaboratory comparisons and surveillance [44,45]. The use of a cut-off value of < 4 loci difference for the assignment of isolates to spatiotemporally related clusters is consistent with previous reports, supporting that further epidemiological investigations are needed to increase the likelihood of correctly assigning isolates to transmission clusters [10,18].

Studies using WGS have contributed considerably to the understanding of VRE transmission within and across healthcare institutions [46]. Healthcare-associated outbreaks of VREfm affecting single institutions or healthcare networks have been reported from several European countries such as Germany, Ireland and the Netherlands [3,10,47]. However, only a few studies, investigated an interregional spread of clonally-related VREfm isolates to geographically distant hospitals [44,48,49], most of which applied a less discriminative sequencing method and investigated mainly clinical samples. Although, we acknowledge that our study largely lacked information on environmental sampling within individual facilities to assess the presence of unrecognised reservoirs.

Dealing with the spread of an antibiotic-resistant organism in a centralised manner poses a number of challenges. First, the level of local prevention and control measures, particularly the rigour of active surveillance cultures and the thoroughness of contact investigations, varies greatly. From a centralised perspective, it is almost impossible to determine whether increased detection of local cases is due to an increased influx from another hospital or to local transmissions as a result of deficient local IPC measures. National recommendations are therefore perceived as exaggerated and of questionable benefit, and as such may be poorly implemented due to the high financial and human resource costs involved. Adapting them to local needs requires IPC teams with sufficient experience and institutional reputation. Second, although the reporting of VRE outbreaks has been mandatory in Switzerland since 2020, this measure is not sufficient to warn other hospitals in time due to delays in reporting. As updates on the initial outbreak situation are voluntary, they are rarely provided. Absence of mandatory WGS and timely exchange of this information between healthcare institutions is another major shortcoming. The delay between the first occurrence of cases and the detection of links between facilities and regions challenges the timely implementation of control measures.

In addition, we caution readers that, owing to the multicentric study design and limited funding, we lacked both a clearly defined at-risk population for calculating relative risks and a control group for estimating odds ratios. Therefore, our epidemiological data do not permit the identification of risk factors.

## Conclusion

Inadequate implementation of active surveillance cultures and inconsistent contact tracing were most likely major contributors to the spread of VRE. Furthermore, passive data surveillance, as conducted by ANRESIS, detects signals of regional development of pathogen prevalence, but may be not sufficient to detect an interregional outbreak with a single clone. This study highlights the need for a national centre to investigate nosocomial outbreaks, enabling the collection of national epidemiological data via a central database. Efforts from local hospitals and the NOIC should be coordinated to provide data and disseminate recommendations for prevention and control in a timely manner. Future prevention efforts should emphasise assessing barriers and facilitators for active surveillance cultures, promoting communication standards between healthcare facilities to share information on VRE cases and contacts and issuing national standards to implement cost-effective sequencing methods, sharing them on a united platform along with epidemiological meta-data.

## Data Availability

All sequence reads have been submitted to ENA under the projects number PRJEB80474/ERP16448.

## Note

Parts of this study have been presented as a poster/poster flash presentation at the Joint Annual Meeting 2024 of the Swiss Society of Infectious Diseases, the Swiss Society of Microbiology, and the Swiss Society for Hospital Hygiene (28–30 August 2024, Bern, Switzerland).

## Supplementary Data

186000 Supplementary Material
