# Supplementary material for: Emergence of vancomycin-resistant Enterococcus faecium vanA ST612 with reduced daptomycin susceptibility, Switzerland, 2018 to 2024
Source: Euro Surveill. 2025 Nov 13;30(45):2500227. doi: 10.2807/1560-7917.ES.2025.30.45.2500227 (PMC12633707; doi:10.2807/1560-7917.ES.2025.30.45.2500227)

This supplementary material is hosted by *Eurosurveillance* as supporting information alongside the article “Emergence of vancomycin-resistant *Enterococcus faecium vanA* ST612 with reduced daptomycin susceptibility, Switzerland, 2018 to 2024”, on behalf of the authors, who remain responsible for the accuracy and appropriateness of the content. The same standards for ethics, copyright, attributions and permissions as for the article apply. Supplements are not edited by *Eurosurveillance* and the journal is not responsible for the maintenance of any links or email addresses provided therein.

Supplementary Figure S1. Susceptibility testing for daptomycin by broth microdilution method of the 72 VRE isolates submitted during the intensified screening period from February to March 2024.

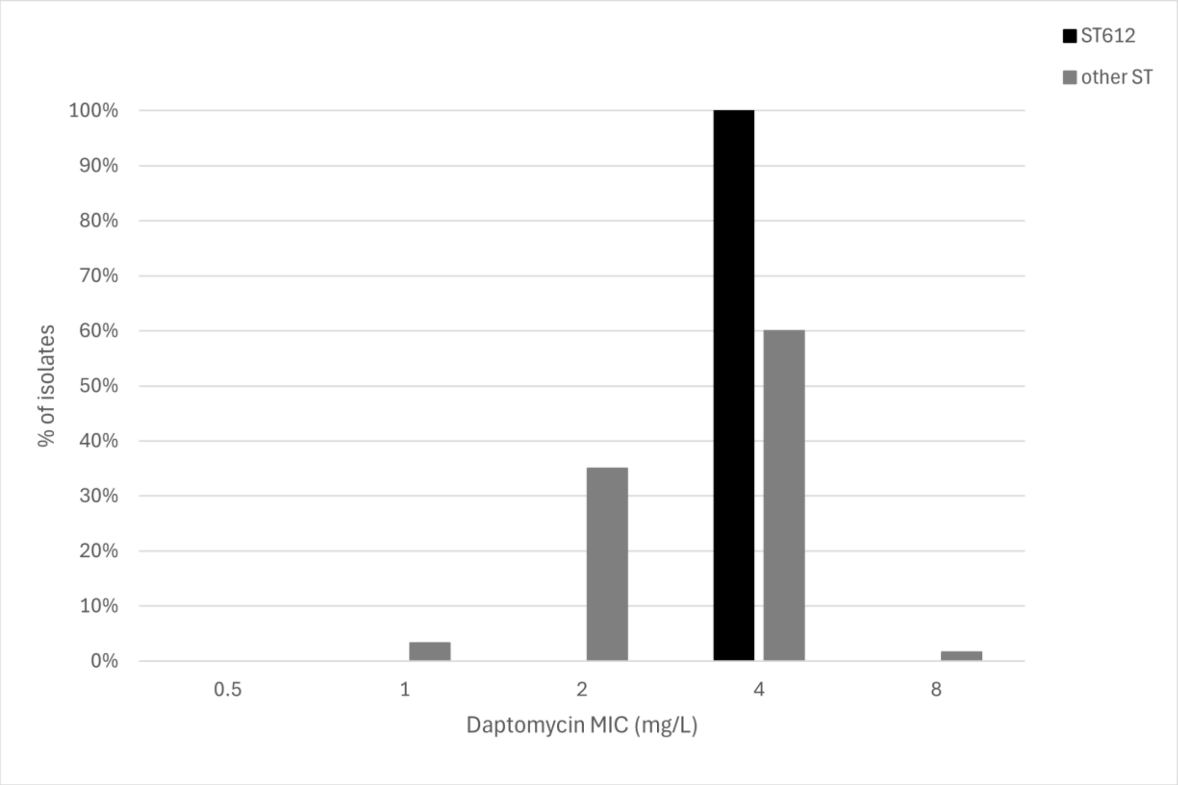

Supplement: Supplementary Material [file 25-00227_VUICHARD-GYSIN_Supplement.pdf]
